# Association Mapping and Functional Analysis of Rice Cold Tolerance QTLs at the Bud Burst Stage

**DOI:** 10.1186/s12284-021-00538-0

**Published:** 2021-11-26

**Authors:** Dan Wang, Zhuo Liu, Yinghui Xiao, Xionglun Liu, Yue Chen, Zhuo Zhang, Houxiang Kang, Xuli Wang, Su Jiang, Shasha Peng, Xinqiu Tan, Deyong Zhang, Yong Liu, Guo-Liang Wang, Chenggang Li

**Affiliations:** 1 College of Agronomy, Hunan Agricultural University, Changsha, 410128 Hunan China; 2 State Key Laboratory of Hybrid Rice and Institute of Plant Protection, Hunan Academy of Agricultural Sciences, Changsha, 410125 China; 3 Department of Plant Pathology, The Ohio State University, Columbus, 43210 USA; 4 State Key Laboratory for Biology of Plant Diseases and Insect Pests, Institute of Plant Protection, Chinese Academy of Agricultural Sciences, Beijing, 100193 China

**Keywords:** Rice, Cold tolerance, Bud burst stage, Genome-wide association study

## Abstract

Cold tolerance at the bud burst stage (CTB) is a key trait for direct-seeded rice. Although quantitative trait loci (QTL) affecting CTB in rice have been mapped using traditional linkage mapping and genome-wide association study (GWAS) methods, the underlying genes remain unknown. In this study, we evaluated the CTB phenotype of 339 cultivars in the Rice Diversity Panel II (RDP II) collection. GWAS identified four QTLs associated with CTB (*qCTBs*), distributed on chromosomes 1–3. Among them, *qCTB-1-1* overlaps with *Osa-miR319b*, a known cold tolerance micro RNA gene. The other three *qCTBs* have not been reported. In addition, we characterised the candidate gene *OsRab11C1* for *qCTB-1-2* that encodes a Rab protein belonging to the small GTP-binding protein family. Overexpression of *OsRab11C1* significantly reduced CTB, while gene knockout elevated CTB as well as cold tolerance at the seedling stage, suggesting that *OsRab11C1* negatively regulates rice cold tolerance. Molecular analysis revealed that *OsRab11C1* modulates cold tolerance by suppressing the abscisic acid signalling pathway and proline biosynthesis. Using RDP II and GWAS, we identified four *qCTBs* that are involved in CTB and determined the function of the candidate gene *OsRab11C1* in cold tolerance. Our results demonstrate that OsRab11C1 is a negative regulator of cold tolerance and knocking out of the gene by genome-editing may provide enhanced cold tolerance in rice.

**Supplementary Information:**

The online version contains supplementary material available at 10.1186/s12284-021-00538-0.

## Background

Rice (*Oryza sativa* L*.*), a major food resource for humans, grows in tropical to temperate regions worldwide. Cold stress is one of the environmental restrictions that most strongly influences rice growth and development, especially at the early seedling and reproductive stages (Liu et al. 2018). At the early seedling stage, low temperature leads to reduced germination, weak seedling establishment, and subsequent yield loss (Yang et al. 2020a). Direct-seeded rice (DSR), a labour-saving and efficient method, has been adopted in many rice growing areas. However, cold stress at the germination and bud burst stages is a major limitation for DSR due to high sensitivity to cold at these stages (Yang et al. 2020b). Improving low-temperature germinability (LTG) and cold tolerance at the bud burst stage (CTB) in rice is the most economical and effective solution to this problem.

Both LTG and CTB are complex genetic traits controlled by multi-genes or quantitative trait loci (QTL). Traditional linkage mapping and genome-wide association study (GWAS) approaches have identified more than 60 LTG-related QTLs and 40 CTB-associated QTLs (Liu et al. 2018; Yang et al. 2020a). To date, only *qLTG3-1* and *OsSAP16* have been cloned and functionally characterised from two identified QTLs for LTG in rice (Fujino et al. 2008; Wang et al. 2018). The QTL gene *qLTG3-1*, encoding a protein of unknown function, is conserved in many plants and is highly expressed in the embryo, which may accelerate vacuolation to weaken tissues covering the embryo, and promote seed germination (Fujino et al. 2008). The rice zinc finger domain protein OsSAP16 (Stress-Associated Protein 16) is the causal gene of the *QTL qLVG7-2* (Wang et al. 2018); loss of *OsSAP16* function reduces germination, while overexpression enhances germination under low temperature.

Previous studies suggest that small GTP-binding proteins (G-proteins) may be involved in the pathway regulating cold tolerance in rice (Nahm et al. 2003; Chen et al. 2011; Xu and Cai 2014). The Rab-type small G-protein OsRAB7 functions in response to cold stress by altering expression in rice (Nahm et al. 2003). Ran-type small G-proteins OsRNA1 and OsRNA2 promote the assembly of the nuclear envelope to avoid damage from cold stress (Chen et al. 2011; Liu et al. 2018). The cloned *COLD1* gene confers chilling tolerance in japonica rice at the seedling stage and encodes a plasma membrane-localised regulator of G-protein signalling (Ma et al. 2015).

Due to the availability of high-density single-nucleotide polymorphism (SNP) maps and diverse rice germplasm resources, GWAS has become an established strategy for efficient and high-throughput gene identification in rice (Wang et al. 2020). Based on Rice Diversity Panel I (RDP I, consisting of 413 cultivars collected from 82 countries), GWAS has been performed on various rice traits (Zhao et al. 2011; Kang et al. 2016; Wang et al. 2018). Our previous GWAS results based on RDP I revealed 67 cold tolerance QTLs at the young seedling stage, and a novel candidate cold resistant gene, *Osryh1*, was identified (Wang et al. 2016). More recently, RDP II has been developed, providing a higher-density dataset of 700K SNPs, and this has been applied to investigate disease resistance (rice blast and rice black-streaked dwarf virus disease) and agronomic characters (tillering) using GWAS (McCouch et al. 2016; Feng et al. 2019; Jiang et al. 2019; Liu et al. 2020). Therefore, GWAS using high-density SNPs within the RDP II population holds promise for dissecting the genetic architecture of a variety of complex traits in rice.

As yet, no causal genes for QTLs of CTB (*qCTBs*) have been identified, hence the genetic basis and underlying molecular mechanism remain poorly understood. In the present study, we performed GWAS using the 339 RDP II cultivars (McCouch et al. 2016) to investigate CTB in rice. We identified four *qCTBs* strongly associated with CTB variation, distributed on chromosomes 1–3. Among them, *qCTB-1-1* overlaps with the microRNA gene *Osa-miR319b* that has a known function in cold tolerance. The other three qCTBs have not been reported.

In addition, we characterised the candidate gene *OsRab11C1* for *qCTB-1-2* (Pitakrattananukool et al. 2012), a Rab-type G-protein that is highly conserved in partially tolerant rice cultivars. Overexpression of *OsRab11C1* significantly reduced CTB, while gene knockout elevated CTB as well as cold tolerance at the seedling stage. These results provide new insight into G-protein function in the CTB pathway, and a candiate gene for engineering cold tolerant rice cultivars via genome editing.

## Results

### CTB Evaluation of the RDP II Population

**Fig. 1:**
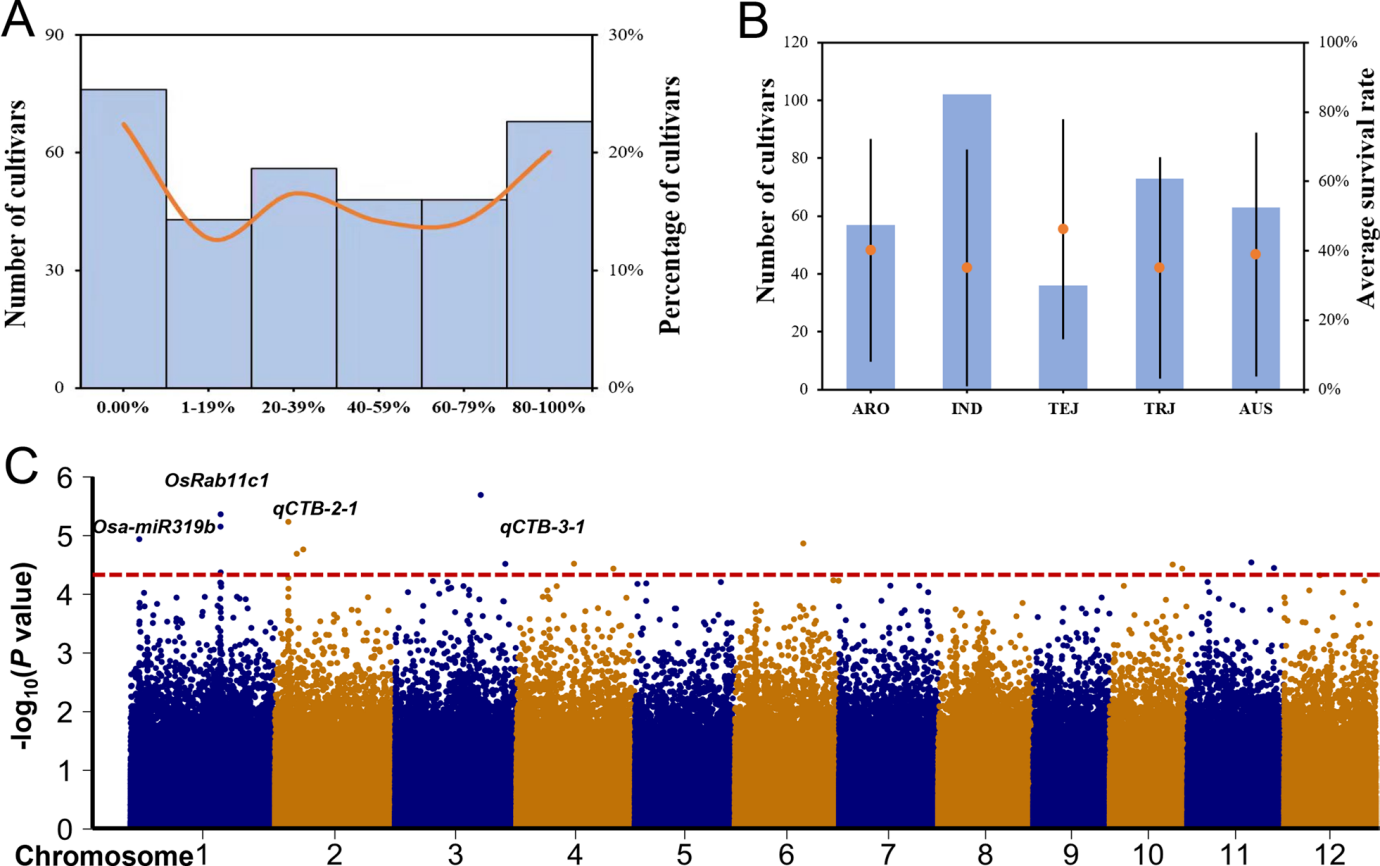
Cold tolerance at the bud burst stage (CTB) of the RDP II population and GWAS of QTLs. **A** Distribution of the survival rate of 339 cultivars after cold treatment. The x-axis shows survival rate and the y-axis represents the number of cultivars. **B** Rice cultivars numbers (left y-axis) of each subgroup (x-axis) used in the study, and the average survival rate (right y-axis) with error bars for each subgroup. **C** Manhattan plots of the association between SNPs and CTB phenotypes based on GWAS. The x-axis shows the genomic coordinates and the y-axis represents the LOD score for each SNP

Three hundred and thirty-nine RDP II rice cultivars were evaluated for CTB using survival rate as an index. The population comprised 102 indica (IND), 22 temperate japonica (TEJ), 88 tropical japonica (TRJ), 56 aromatic (ARO), 64 aus (AUS), and 7 admixture (ADM) cultivars, and their CTB phenotypes are shown in Additional file 1: Table S1. The number of cultivars with survival rate between 0, 1–19, 20–39, 40–59, 60–79 and 80–100% was 76, 43, 56, 48, 48 and 68, respectively (Fig. 1A). Comparison of CTB for the five major sub-populations revealed an average survival rate for the 339 cultivars was ~ 0.41, and the average survival rate of TEJ, ARO, IND, TRJ and AUS was 0.46, 0.40, 0.35, 0.35 and 0.39, respectively (Fig. 1B). The ADM group was not included because it only contained seven cultivars and the variation of the survival rate among the cultivars was large. Among the 339 cultivars, there were 40 highly tolerant cultivars (survival rate 90–100%; Additional file 2: Table S2). Among them, 11, 9, 4, 10, 4 and 2 belonged to sub-populations of IND, TRJ, ARO, AUS, TEJ and ADM, respectively. The percentage of the highly tolerant cultivars in IND, TRJ, ARO, AUS, TEJ and ADM was 29.3%, 22%, 9.7%, 24.5%, 9.7% and 4.8%, respectively. Interestingly, 25 of the highly tolerant cultivars were collected from Asian countries (Additional file 2: Table S2), including Bangladesh (8), Philippines (5), India (4), China (3), Pakistan (2), Thailand (1), Sri Lanka (1) and Nepal (1). These highly tolerant cultivars should be excellent breeding materials for rice cold tolerance breeding programs.

### GWAS of *qCTBs*

To map the QTLs corresponding to *qCTBs*, we performed GWAS using the CTB phenotypes and the high-density SNP datasets of the 339 rice cultivars. Seventeen significantly associated SNPs [− Log_10_(*p*-value) ≥ 4.3] were detected (Fig. 1C; Additional file 3: Table S3). Any locus bracketed by two significantly associated SNPs within a 200 kb interval region was considered a *qCTB*. The analysis identified four *qCTBs* in the rice genome (Additional file 4: Table S4). To identify candidate genes, we analysed a 200 kb genomic region of the Nipponbare (NPB) reference genome for each *qCTB* and selected genes such as those known to be related to cold responses as candidates. A total of 138 candidate genes were predicted for the four *qCTBs* (Additional file 4: Table S4). Among them, *qCTB-1-1* overlapped with *Osa-miR319b* (Wang et al. 2014). The remaining three *qCTBs* were identified for the first time in this study.

### *OsRab11C1* is a Candidate Gene for *qCTB-1-2*

Two significant SNPs (LOD > 4.3) at the *qCTB-1-2* locus on chromosome 1 were closely associated with cold tolerance in 10 highly cold tolerant and 10 cold sensitive cultivars (Additional file 5: Table S5)*.* Within the 200-kb region, the candidate gene, *LOC_Os01g47730,* designated *OsRab11C1,* encodes a Rab-type small G-protein (Pitakrattananukool et al. 2012). Small G-proteins, OsRAN1, OsRAN2 and OsRYH1, functioning in cold tolerance have been reported previously (Chen et al. 2011; Xu and Cai 2014; Wang et al. 2016). Thus, *OsRab11C1* was considered an ideal candidate gene for *qCTB-1–2*.

**Fig. 2:**
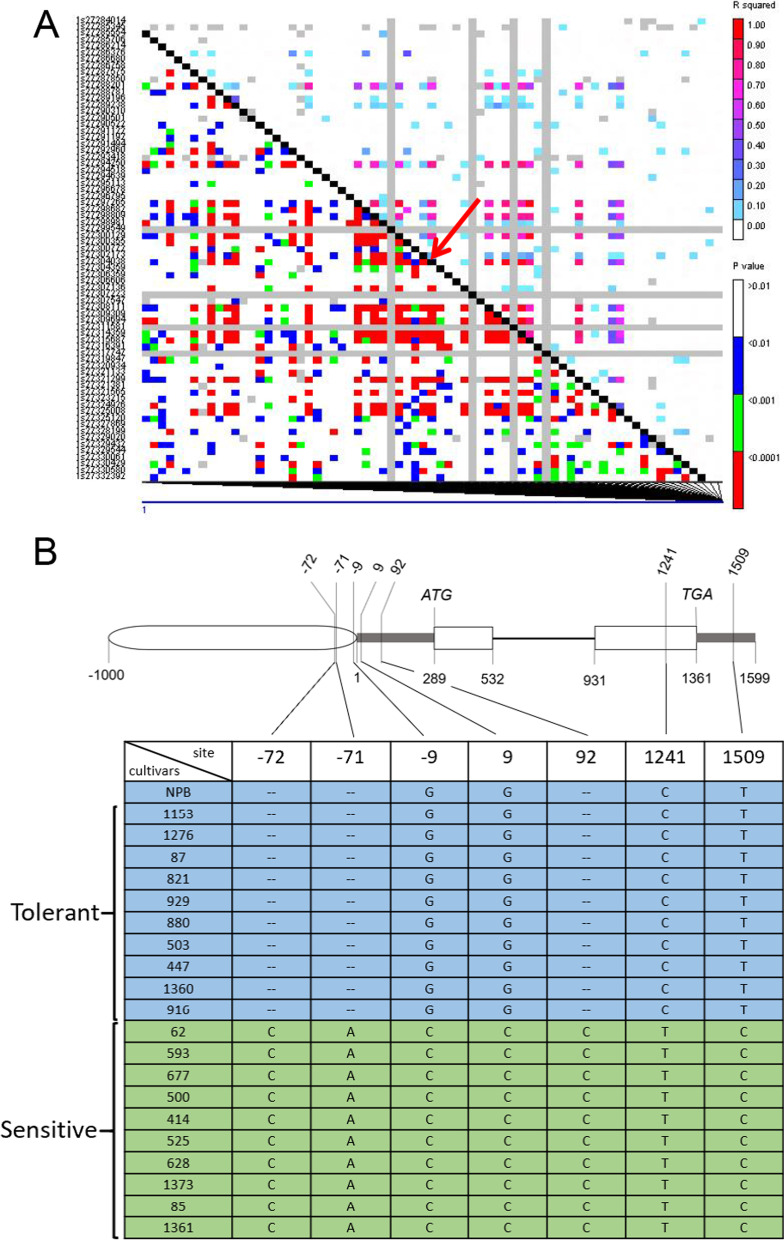
Sequence polymorphism between cold tolerant and sensitive cultivars for *OsRab11C1*. **A** LD map for the 19.1 kb region containing *OsRab11C1* (indicated by the red arrow). Triangle plot for pairwise values (r^2^) were plotted against physical genomic distantance between markers in the 19.1 kb region. The pairwise LD values of polymorphic SNPs are plotted on both the x-and y-axes. Each cell represents a comparison of two pairs of marker sites and the cells are color-coded with respect to the presence of significant LD. A colored barcode for the significance threshold levels in both diagonals is shown. **B** Sequence analysis of the candidate gene *OsRab11C1* in rice cultivars with extremely low and high survival rates. Tilted numbers at the top represent the position related to the initiation codon of the 5’-UTR, ‘−’ indicates the upstream region of the 5’-UTR, and blank rectangles indicate gene exon regions. The table shows sequence variation between the two groups of cultivars. A, T, G and C are the nucleotide bases, and ‘–’ indicates deletion

To confirm the association between *OsRab11C1* and *qCTB-1–2*, we constructed a linkage disequilibrium (LD) map for 200 kb region of *OsRab11C1,* which showed the SNP-1.27301128 (LOD = 0.08) locating in *OsRab11C1* was not associated with *qCTB-1-2* (Fig. 2A)*.* Next we sequenced *OsRab11C1* in the genomic DNA of 10 highly cold-tolerant and 10 cold-sensitive cultivars. The results revealed seven mutations (deletions and insertions) in the promoter and coding regions of *OsRab11C1* between cold-tolerant and cold-sensitive cultivars (Fig. 2B). The only SNP, at position 1241 bp in the second exon of *OsRab11C1*, changes the amino acid from alanine (A) in cold-tolerant cultivars to valine (V) in cold-sensitive cultivars, which may affect gene/protein function. These results implicated *OsRab11C1* as a strong candidate gene for *qCTB-1-2*.

### *OsRab11C1* Expression Pattern and Sub-cellular Localization

**Fig. 3:**
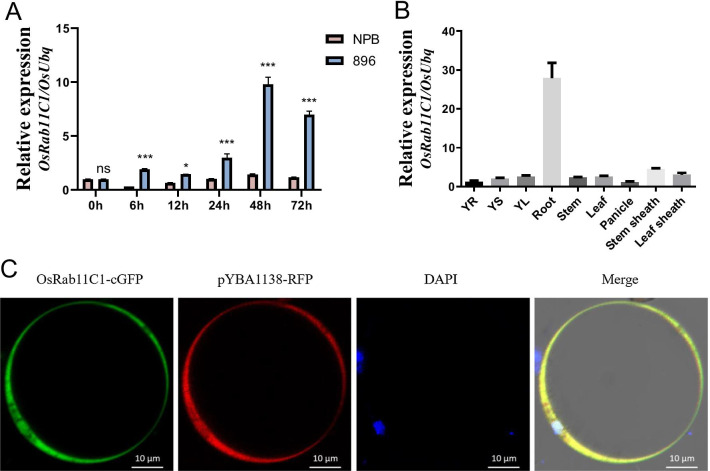
Expression analysis and subcellular localisation of *OsRab11C1.* **A** Relative expression levels of *OsRab11C1* in Nipponbare (NPB) and the extremely sensitive cultivar 896 after cold treatment at 4 °C. Pink bars show expression levels of *OsRab11C1* in Nipponbare and blue bars are expression levels in 896. The y-axis represents relative gene expression levels. “ns, * and ***” indicate no significant differences, significant differences (*p* = 0.05), and highly significant differences (*p* = 0.001), respectively. **B** Relative expression levels of *OsRab11C1* in different rice tissues. The x-axis represents different rice tissues. YR, young roots; YS, young stems; YL, young leaves. **C** Subcellular localisation of OsRab11C1 in rice protoplasts. pYBA1138-RFP was used as a whole-cell marker. DAPI (4′,6-diamidino-2-phenylindole) was used to stain nuclei

Next, we investigated the *OsRab11C1* expression pattern under cold treatment. We performed a quantitative real-time PCR (qRT-PCR) assay using total RNA isolated from seedlings of cold-tolerant cultivar NPB and cold-sensitive cultivar 896 (“JUMA” in the *aus* subgroup from Bangladesh) sampling at 0, 6, 12, 24, 48 and 72 h after 4 °C cold treatment. As shown in Fig. 3A, expression of *OsRab11C1* was not altered significantly in NPB, but was strongly induced in 896. This result is consistent with that from genetic analysis showing that *OsRab11C1* is a negative regulator of rice cold tolerance described below. To investigate the tissue specificity of *OsRab11C1* gene expression, we also probed the expression of *OsRab11C1* in nine different tissues and found that *OsRab11C1* was highly expressed in roots (Fig. 3B). According to the GenScript website (https://www.genscript.com/wolf-psort.html), OsRab11C1 was predicted to be mainly localized in the cytoplasm. To confirm this, we cloned the *OsRab11C1* coding region from NPB into the pYBA1132-GFP (green fluorescent protein) vector and transfected the OsRab11C1-pYBA1132-GFP construct into rice protoplasts of NPB. The results show that OsRab11C1-GFP was co-localized with red fluorescent protein (RFP) in the entire rice cell (Fig. 3C).

### *OsRab11C1* Negatively Regulates Rice Cold Tolerance

**Fig. 4:**
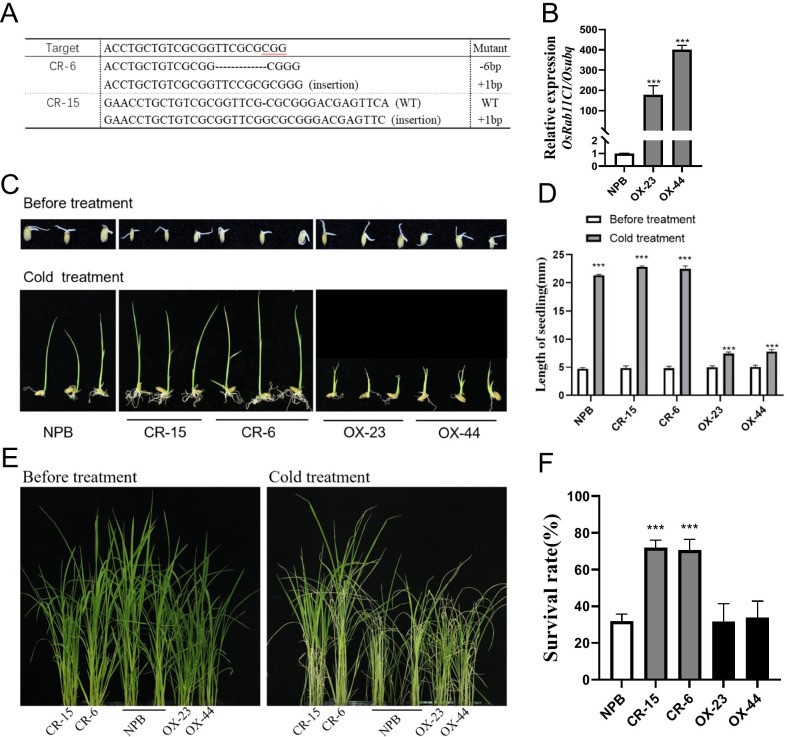
Assessment of cold tolerance in *OsRab11C1* knockout mutants and overexpression transgenic plants. **A** Mutations in the two deletion mutants of *OsRab11C1* in the Nipponbare (NPB) background using CRISPR-Cas9 (CR-6, CR-15). **B** The two overexpression mutants of *OsRab11C1* in the NPB background(OX-23, OX-44). **C** Plant growth of the *OsRab11C1* mutants and NPB under cold treatment at the bud burst stage. **D** The statistical analysis of the bud length of the *OsRab11C1* mutants and NPB under cold treatment at the bud burst stage. **E** Plant growth of the *OsRab11C1* mutants and NPB under cold treatment at the seedling stage. **F** Evaluation of the seedling survival rate of the *OsRab11C1* overexpression and knockout mutants and NPB under cold treatment at the seedling stage

To validate whether *OsRab11C1* does indeed control rice cold tolerance, we knocked out *OsRab11C1* (*OsRab11C1-*KO) in the NPB background using the CRISPR/Cas9 (Clustered Regularly Interspaced Short Palindromic Repeats/CRISPR-associated protein 9) gene editing system. The guide RNA (gRNA) is located at position 102–123 bp in the coding region of *OsRab11C1.* We sequenced the gRNA targeted region and selected two T_0_ plants (CR-6 and CR-15) containing insertions and deletions in the target region for subsequent phenotype analysis (Fig. 4A). We also generated overexpression lines of *OsRab11C1* (*OsRab11C1-*OE) under the control of the maize ubiquitin promoter in the NPB background. The relative expression level of *OsRab11C1* in the two overexpression lines (OX-23 and OX-44) were 177- and 400-fold higher than that in NPB, respectively (Fig. 4B). We used homozygous T_1_ plants of CR-6, CR-15, OX-23 and OX-44 for cold tolerance evaluation. At the bud burst stage under cold stress, the growth of the knockout mutants CR-6 and CR-15 was better than that of wild-type (WT) NPB plants, but not statistically different (Fig. 4C and 4D). By contrast, OX-23 and OX-44 overexpression plants grew quite slowly after cold treatment (Fig. 4C and 4D). Interestingly, both CR-6 and CR-15 seedlings showed stronger cold tolerance than NPB and overexpression seedlings after cold treatment (Fig. 4E), and the seedling mortality of CR-6 and CR-15 was only ~ 30% compared with 60–70% for NPB, OX-23 and OX-44 (Fig. 4F). These results indicate that *OsRab11C1* negatively regulates cold tolerance in rice.

### Suppression of Abscisic Acid (ABA) Signalling and Proline Biosynthesis Pathways by OsRab11C1

**Fig. 5:**
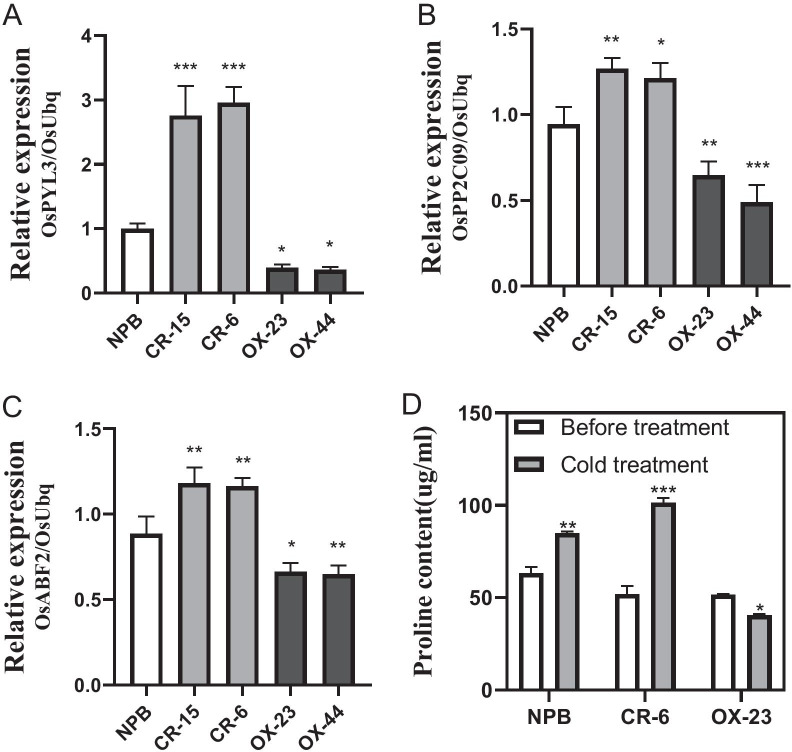
Expression analysis of the three ABA-related genes and proline content in *OsRab11C1* transgenic lines. Relative gene expression levels of *OsPYL3* (**A**), *OsABF2* (**B**) and *OsPPC09* (**C**) in *OsRab11C1* transgenic lines. The X-axis represents the knockout lines (CR-6, CR-15), the overexpression lines (OX-23, OX-44) and wild-type Nipponbare (NPB). The Y-axis shows relative gene expression. **D** Proline content of the *OsRab11C1* transgenic and NPB lines after 4 °C cold treatment

To further explore the mechanism of *OsRab11C1*-mediated cold responses, we measured the expression levels of three marker genes (*OsPYL3*, *OsABF2* and *OsPPC09*) in *OsRab11C1-*OE and knockout mutants, and WT plants, using qRT-PCR. These genes are critical for ABA signal transduction under cold stress (Hossain et al. 2010; Chen et al. 2014; Tian et al. 2015). The results showed that the relative expression levels of *OsPYL3*, *OsABF2* and *OsPPC09* in *OsRab11C1-*OE plants were significantly down-regulated, while they were up-regulated in *OsRab11C1* mutant plants, compared with those in WT plants (Fig. 5A–C), suggesting that *OsRab11C1* negatively regulates cold responses by suppressing the ABA signalling pathway in rice.

We also measured the levels of proline, a molecule that protects plants against cellar damage. As shown in Fig. 5D, the average proline content in 2-week-old seedlings of WT, mutant CR-6 and OX-23 plants under normal growth conditions (25 °C) were 63.20, 51.87 and 51.53 μg/ml, respectively. After 4 °C cold treatment, the average proline content in CR-6 plants was increased to 101.34 μg/ml, significantly higher than in WT plants (84.80 μg/ml). By contrast, the average proline content in OX-23 plants was decreased to 40.43 μg/ml, much lower than in WT plants. These results suggest that *OsRab11C1* also negatively regulates proline biosynthesis in rice.

## Discussion

As climate change advances, cold stress at the seedling stage is becoming a major challenge for both indica and japonica rice production in many countries. To save labour and reduce cost, direct seeding is replacing traditional puddle transplanting in small farms. However, the low germination rate and slow seedling growth of most modern rice cultivars severely hinders the wide application of this technique in Asia and Africa. One of the reasons is the lack of availability of rice varieties for direct seedling with strong cold tolerance. In this study, we identified 40 highly tolerant varieties from all subgroups in RDP II exhibiting at least 90% survival rate under cold treatment at the bud burst stage. Among the 16 varieties with 100% survival rate, most (10) are from Asian countries such as Bangladesh and the Philippines, and four are from South American countries including Brazil and Columbia. These varieties can be directly used as parental lines in genetic crosses in local breeding programs. Therefore, our study has provided valuable cold tolerance materials for both indica and japonica rice breeding programs.

Among the four *qCTBs* identified in this study, *qCTB-1-1* is co-localised with known cold tolerance gene *Osa-miR319b* (Wang et al. 2014). The other three *qCTBs* are newly discovered cold tolerance QTLs. These loci are candidates for further genetic analysis to determine their roles in rice cold tolerance. In addition, DNA markers could be developed from these loci for molecular breeding programs.

We genetically characterised the Rab small G-protein gene *OsRab11C1*, which is the causal gene of *qCTB-1-2* for rice cold tolerance. Previous research demonstrated that other small G-protein genes including *OsRAN1*, *OsRAN2* and *OsRYH1* regulated cold tolerance in rice (Chen et al. 2011; Xu and Cai 2014; Wang et al. 2016). Interestingly, unlike these genes that act as positive regulators for rice cold tolerance, *OsRab11C1* plays a negative role for CTB, expanding the functions of GTPase-mediated cold tolerance in rice. Whether the function of *OsRab11C1* in other plants is conserved remains to be investigated. In addition, it will be interesting to identify the regulator(s) of OsRab11C1 in cold signalling transduction. COLD1 interacts with the G-protein α subunit to activate the Ca^2+^ channel to sense low temperature and accelerate G-protein GTPase activity (Ma et al. 2015; Zhu 2016). It remains to be determined if COLD1 can interact with OsRab11C1 in rice cells.

ABA is a key phytohormone and signalling molecule in response to cold stress in plants (Cutler et al. 2010; Zhu 2016; Liu et al. 2018). Several genes involved in ABA signal transduction, such as *OsPYL3*, *OsABF2* and *OsPP2C09*, are important for plant cold tolerance (Hossain et al. 2010; Chen et al. 2014; Tian et al. 2015). Relationships between Rab proteins and ABA signalling have not been clearly demonstated. Herein, we found that the expression levels of *OsPYL3*, *OsABF2* and *OsPP2C09* were much higher in knockout plants and much lower in overexpression plants than in WT plants, suggesting that *OsRab11C1* may play an important role in the ABA-dependent pathway during cold stress. Whether OsRab11C1 interacts with rice ABA receptors such as OsPYLs, and how OsRab11C1 represses ABA signalling gene activation, remain to be investigated.

## Conclusions

Cold tolerance is one of the major challenges in rice production in many rice growing regions. From the RDP II population, we identified 40 highly tolerant rice cultivars with survival rate over 90% after cold treatment at the bud burst stage. Using the cold phenotypes of RPD II and GWAS, we identifyed four *qCTBs*. Among them, *qCTB-1-1* ais co-localized with known cold-associated gene and the other three have not been reported..We further characterised the candidate gene *OsRab11C1* for *qCTB-1-2*, which encodes a Rab-type small GTP-binding protein. Overexpression of the gene significantly reduces CTB, while gene knockout elevates CTB as well as cold tolerance at the seedling stage. Molecular analysis indicates that *OsRab11C1* modulates cold tolerance by suppressing the ABA signalling pathway. Our results demonstrate that *OsRab11C1* negatively regulates rice cold tolerance and can be used for enhancing rice cold tolerance at the early stages via genome-editing or marker-aided selection.

## Materials and Methods

### Plant Materials

The 339 cultivars from the RDP II population are comprised of six sub-populations (102 IND, 22 TEJ, 88 TRJ, 56 ARO, 64 AUS, and 7 ADM). The 700K SNP dataset of the 339 RDP II cultivars was generated from a previous study (McCouch et al. 2016).

### Evaluation of CTB

The cold tolerance score was defined by the survival rate which is the percentage of rice seedlings survived from the bud stage to the seedling stage after cold treatment following the formula: cold tolerance (%) = number of seedlings/number of buds × 100) (Han et al. 2004). About 25 seeds of each cultivar were germinated in Petri dishes in an incubator at 37 °C for three days. The germinated seeds were then transferred into a growth chamber for one day at 25 °C with a relative humidity (RH) of 75% and a 14 h light and 10 h dark photoperiod. Germinated rice seeds were subjected to cold treatment at 4 °C with the same relative humidity and photoperiod for seven days. After seven days of exposure to cold stress, the cold tolerance scores of the 339 rice cultivars were calculated. The experiment was repeated three times under the same environment, and the average cold tolerance scores from three replicates were used for GWAS.

### GWAS of Rice Cold Tolerance at the Bud Burst Stage

Like our previous GWAS on rice blast, the 416,065 SNPs of 339 RDP II accessions were generated from the 700K SNP RDP II genotypes using P-link with the criterion of minor allele frequency ≥ 5%. GWAS was performed and Manhattan and Q-Q plots were generated. SNPs with − Log_10_(*P*-value) ≥ 4.3 − log_10_(1/M) = 4.3, (M = 416,065) were considered to be significant association (Li et al. 2019).

### Identification of QTLs and Selection of Cold Tolerance Candidate Genes

QTLs were identified in the 200 kb interval regions using the Nipponbare genome as a reference. Candidate genes around the peak SNPs were selected, and all of the reported temperature-related genes in plants were assumed to be candidate genes (Wang et al. 2016).

### Sequence Analysis of Genomic DNA from *OsRab11C1*

The full-length sequence, from − 2000 bp upstream of the 5’-untranslated region (UTR) to + 1000 bp downstream of the 3’-UTR of *OsRab11C1*, was downloaded from the Rice Genome Annotation Project website (http://rice.plantbiology.msu.edu/analyses_search_blast.shtml). Gene-specific primers were designed for cloning *OsRab11C1* from cultivars that were highly cold tolerant and highly cold sensitive. DNA sequencing of the cloned genes was performed by Sangon Biotech Company (Wuhan, China). Sequence alignment was performed with SeqMan (http://seqman.software.informer.com/). All primers used in the study are shown in Additional file 6: Table S6.

### Gene Expression Analysis

For analysis of the expression pattern and tissue specificity of *OsRab11C1*, total RNA was extracted from root, coleoptile and leaf tissues collected from three seeds of each cultivars using an RNA extraction kit (Promega Biotech, http://www.promega.com.cn). First-strand cDNA synthesis was performed using HiScript II QRT SuperMix (Vazyme, http://www.vazyme.com). Gene expression levels were calculated based on analysis of variance (ANOVA) of three technical replicates. The OsUbq gene (LOC_Os03g61970) was included as an internal control. Three independent replicates were performed. Gene-specific primers for *OsRab11C1* are shown in Additional file 6: Table S6.

### Construction of Vectors and Genetic Transformation

The CRISPR/Cas9 vector system was provided by Dr. YG Liu’s laboratory (South China Agriculture University, Guangzhou, China) (Li et al. 2017). The gene-specific targets were designed online (http://skl.scau.edu.cn/primerdesign/) and the target mutation types were analysed on the website (http://skl.scau.edu.cn/dsdecode/). CRISPR constructs were transformed into NPB using an agrobacterium-mediated transformation method (Liu et al. 2020). All transgenic plants and their progeny were examined by PCR amplification using specific primers targeting the gRNA region. All primer sequences for the constructs are listed in Additional file 6: Table S6.

To overexpress *OsRab11C1*, we used the overexpression vector pCAMBIA1300 described previously (He et al. 2018). The 1599 bp full-length coding sequence region was cloned using gene-specific primers from cDNA obtained by reverse transcription using mRNA from NPB seedlings. The resulting gene was cloned into the pCAMBIA1300 vector to generate the overexpression construct, which was transformed into *Agrobacterium tumefaciens* strain EHA105 and subsequently transformed into NPB. All transgenic plants and their progeny were examined by PCR amplification using specific primers.

### Sub-cellular Localization of OsRab11C1

The overexpression construct OsRab11C1-pYBA1132-GFP was transfected into rice protoplasts of NPB following the method reported previously (He et al. 2018).

### Determination of Cellular Proline Levels

Two-week-old seedlings (0.1 g) of NPB and *OsRab11C1* transgenic rice lines (CR-6 and OX-23) with or without 4 °C cold treatment were homogenised in 2 ml of 3% aqueous sulfosalicylic acid using liquid nitrogen. Acid ninhydrin, methylbenzene, and glacial acetic acid were added and samples centrifuged. The free proline content was measured using a spectrophotometer as described previously (Bates et al. 1973) and reported as μg/ml.

## Supplementary Information

Additional file 1: Table S1. Survival rate of 339 RPD II cultivars at the bud burst stage after cold treatment.

Additional file 2: Table S2. List of 41 highly cold tolerant cultivars at the bud burst stage.

Additional file 3: Table S3. List of SNPs significantly associated with CTB.

Additional file 4: Table S4. List of four QTLs related to CTB.

Additional file 5: Table S5. Association of the qCTB1-2-associated significant SNPs in 10 cold tolerant and 10 sensitive rice cultivars.

Additional file 6: Table S6. Primers used in this study.

## Data Availability

The data sets supporting the results of this article are included within the article and its additional files.

## Abbreviations

CTB: Cold tolerance at the bud burst stage
QTLs: Quantitative trait loci
RDP II: Rice Diversity Panel II
*qCTBs*: QTLs associated with CTB
DSR: Direct-seeded rice
LTG: Low-temperature germinability
NPB: Nipponbare
