# Supplementary material for: Association Mapping and Functional Analysis of Rice Cold Tolerance QTLs at the Bud Burst Stage
Source: Rice (N Y). 2021 Nov 26;14:98. doi: 10.1186/s12284-021-00538-0 (PMC8626552; doi:10.1186/s12284-021-00538-0)
Supplement: Supplementary file 2 — List of 41 highly cold tolerant cultivars at the bud burst stage. [file 12284_2021_538_MOESM2_ESM.docx]

**Table S2. List of 41 highly cold tolerant cultivars at the bud burst stage**

| Sub-group | Origin | Acce. No. | Survival rate | Sub-group | Originate | Acce. No. | Survival rate |
| --- | --- | --- | --- | --- | --- | --- | --- |
| AUS | India | 447 | 90.00% | AUS | Bangladesh | 583 | 92.86% |
| IND | Philippines | 464 | 90.00% | AUS | Bangladesh | 24 | 93.10% |
| IND | China | 688 | 90.00% | TEJ | China | 1068 | 93.33% |
| IND | Nepal | 745 | 90.00% | ARO | India | 751 | 95.00% |
| AUS | Bangladesh | 752 | 90.00% | AUS | Bangladesh | 443 | 100.00% |
| IND | Thailand | 767 | 90.00% | IND | Philippines | 527 | 100.00% |
| TRJ | United States | 820 | 90.00% | ARO | Pakistan | 560 | 100.00% |
| ADM | Brazil | 832 | 90.00% | ARO | Bangladesh | 584 | 100.00% |
| AUS | Sri Lanka | 900 | 90.00% | AUS | Pakistan | 659 | 100.00% |
| TEJ | Romania | 923 | 90.00% | IND | China | 684 | 100.00% |
| TRJ | Ivory Coast | 1058 | 90.00% | AUS | Bangladesh | 726 | 100.00% |
| TEJ | Belgium | 1061 | 90.00% | TRJ | Guinea | 810 | 100.00% |
| IND | Madagascar | 1156 | 90.00% | TRJ | Brazil | 880 | 100.00% |
| IND | Philippines | 1268 | 90.00% | TRJ | Ecuador | 981 | 100.00% |
| TRJ | Philippines | 1276 | 90.00% | ARO | India | 1109 | 100.00% |
| TRJ | Colombia | 1321 | 90.00% | AUS | Bangladesh | 1125 | 100.00% |
| IND | Mali | 1342 | 90.00% | IND | Philippines | 1135 | 100.00% |
| ADM | Upper Volta | 1392 | 90.00% | TEJ | Italy | 1320 | 100.00% |
| IND | Bangladesh | 674 | 90.91% | TRJ | Colombia | 1325 | 100.00% |
| AUS | India | 555 | 92.11% | TRJ | Brazil | 1329 | 100.00% |
