# Supplementary material for: Association Mapping and Functional Analysis of Rice Cold Tolerance QTLs at the Bud Burst Stage
Source: Rice (N Y). 2021 Nov 26;14:98. doi: 10.1186/s12284-021-00538-0 (PMC8626552; doi:10.1186/s12284-021-00538-0)
Supplement: Supplementary file 6 — Primers used in this study. [file 12284_2021_538_MOESM6_ESM.docx]

**Table S5. Primers used in the study**

| Primer name | sequence（5'-3') | annotation |
| --- | --- | --- |
| LCTc1-1F | TCCAGGGACGAATTTCAGAC | PCR amplification of *OsRab11C1* |
| LCTc1-1R | GCAGGGGAAGATGGTGAATA | PCR amplification of *OsRab11C1* |
| LCTc1-2F | GACGAGTTCAGCCTGGAGAC | PCR amplification of *OsRab11C1* |
| LCTc1-2R | CGTCTATCTGGGTGGCATTT | PCR amplification of *OsRab11C1* |
| LCTc1-3F | GCACGGGACCTTCTCCAT | PCR amplification of *OsRab11C1* |
| LCTc1-3R | TTTGTTGTGATCCGGGAGT | PCR amplification of *OsRab11C1* |
| CRc1-F | GCCGACCTGCTGTCGCGGTTCGCG | Design CRISPR primer |
| CRc1-R | AAACCGCGAACCGCGACAGCAGGT | Design CRISPR primer |
| Cc1-F | ATGGCGGCGGCGGCGGCG | Clone *OsRab11C1* |
| Cc1-R | TCAAGACGAGCAGCACCCTGAGCT | Clone *OsRab11C1* |
| OEc1-F | CGCGGATCCATGGCGGCGGCGGCGGCG | Construct the carrier of over-expression *OsRab11C1* |
| OEc1-R | CCCAAGCTTTCAAGACGAGCAGCACCCTGAGCT | Construct the carrier of over-expression *OsRab11C1* |
| UBQ2F | CCAGGACAAGATGATCTGCC | cDNA Semi-quantitative analysis |
| UBQ2R | AAGAAGCTGAAGCATCCAGC | cDNA Semi-quantitative analysis |
| UBQQF | CGCAAGAAGAAGTGTGGTCA | qRT-PCR |
| UBQQR | GGGAGATAACAACGGAAGCA | qRT-PCR |
| qRTc1-F | CTTTGACTCGCTCAAACTCATC | Analysis the over-expression of *OsRab11C1* |
| qRTc1-R | AGTATCAATCAGCAGTGACGAA | Analysis the over-expression of *OsRab11C1* |
| CTC1-1F | TAATTGAAGGGAAGCGGTGC | Identification of CRISPR/Cas9 transgenic lines |
| CTC1-1R | CAGAAGCAGGGATGATGGTC | Identification of CRISPR/Cas9 transgenic lines |
| 1132-RabC11-F | GGCCGCTCTAGAACTAGTGGAATGGCGGCGGCGGCGGCGGC | Construct the carrier of sub-cellular localization *OsRab11C1* |
| 1132-RabC11-R | GACGGTATCGATAAGCTTGATAGACGAGCAGCACCCTGAGC | Construct the carrier of sub-cellular localization *OsRab11C1* |
| QRT-OsPP2C09-F | GAGAAAATCTAGCGAGCCGTC | Analysis of the relative expression level of marker gene |
| QRT-OsPP2C09-R | GGCAAGCAACTACTCCAATTAC | Analysis of the relative expression level of marker gene |
| QRT-OsABF2-F | CTTCAGAGTTGCTTCTCTCCTT | Analysis of the relative expression level of marker gene |
| QRT-OsABF2-R | GAACTGTAAACGGGATGACATG | Analysis of the relative expression level of marker gene |
| QRT-OsPYL3-F | CTTTTCAAGCCATTTGTGAGCC | Analysis of the relative expression level of marker gene |
| QRT-OsPYL3-R | GCAGGCCAGACTTAACATTAAC | Analysis of the relative expression level of marker gene |
